# miR-125a-5p impairs endothelial cell angiogenesis in aging mice via RTEF-1 downregulation

**DOI:** 10.1111/acel.12252

**Published:** 2014-07-24

**Authors:** Peng Che, Jun Liu, Zhen Shan, Ridong Wu, Chen Yao, Jin Cui, Xiaonan Zhu, Junwei Wang, Mary Susan Burnett, Shenming Wang, JinSong Wang

**Affiliations:** 1 Department of Vascular Surgery, The First Affiliated Hospital of Sun Yat-sen UniversityNO. 58 Zhongshan Road 2, Guangzhou, 510080, China; 2 Department of Cardiology, The First Affiliated Hospital of Sun Yat-Sen UniversityNO. 58 Zhongshan Road 2, Guangzhou, 510080, China; 3 Department of Pharmacology Laboratory, The First Affiliated Hospital of Sun Yat-sen UniversityNO.58 Zhongshan Road 2, Guangzhou, 510080, China; 4 Cardiovascular Research Institute, MedStar Health Research InstituteWashington, DC, 20010, USA

**Keywords:** aging, angiogenesis, endothelial cell, RTEF-1, miRNA-125a-5p

## Abstract

Increasing evidence suggests that microRNAs (miRNAs) play important roles in impaired endothelial cell (EC) angiogenesis during aging. However, their exact roles in the aging process remain unclear. We aimed to determine whether miRNAs cause angiogenesis defects in ECs during aging and to uncover the underlying mechanisms. To study the miRNA-induced changes in ECs during aging, we performed microarray analyses on arterial ECs collected from young and aging mice. Using qRT–PCR, we showed that microRNA-125a-5p (mir-125a-5p) expression was approximately 2.9 times higher in old endothelial cells (OECs) compared with samples collected from young animals. Western blot assays showed a lower expression level of an mir-125a-5p target known as related transcriptional enhancer factor-1 (RTEF-1) in OECs compared with its expression levels in young cells. Overexpression of mir-125a-5p in young endothelial cells (YECs) using pre-mir-125a-5p caused the downregulation of RTEF-1, endothelial nitric oxide synthase (eNOS) and vascular endothelial growth factor (VEGF) and resulted in impaired angiogenesis, as evidenced by spheroid sprouting and tube formation assays *in vitro*. Conversely, repression of mir-125a-5p in OECs using anti-mir-125a-5p increased RTEF-1, eNOS and VEGF expression and improved EC angiogenesis. Importantly, impaired angiogenesis caused by knock-down of RTEF-1 was not efficiently rescued by anti-mir-125a-5p. Dual-luciferase reporter gene analysis showed that RTEF-1 is a direct target of mir-125a-5p, which regulates angiogenesis by repressing RTEF-1 expression and modulating eNOS and VEGF expression. These findings indicate that mir-125a-5p and RTEF-1 are potential therapeutic targets for improving EC-mediated angiogenesis in elderly individuals.

## Introduction

Aging is considered an independent risk factor for the development of peripheral vascular disease (Rivard *et al*., 1999), and the adaptive growth of blood vessels is an important protective factor against this disease. Endothelial cells (ECs) play a crucial role in adaptive blood vessel growth (Sadoun & Reed, 2003). Recent studies have demonstrated the attenuation of pre-existing small collateral arteries and capillary sprouting in lower limbs in aged animals as well as decreased expression of endothelial nitric oxide synthase (eNOS), vascular endothelial growth factor (VEGF) and other cytokines in ECs that contribute to impaired angiogenesis during aging (Carmeliet, 2000; Grundmann *et al*., 2011).

Recently, microRNAs (miRNAs) have been identified as important regulators of gene expression in a wide range of organisms and biological systems (Ambros, 2004). miRNAs incorporate into the RNA-induced silencing complex, which then recognizes the sequences of target mRNAs by imperfect base pairing, thereby regulating the expression of these genes by translational repression (Sadoun & Reed, 2003). Certain miRNAs are involved in the decline of EC function during aging (Menghini *et al*., 2009; Zhao *et al*., 2010). However, the relationship between miRNAs and impaired angiogenic proteins in aging ECs has not been fully defined.

Related transcriptional enhancer factor-1 (RTEF-1) is a member of the transcriptional enhancer factor (TEF) family, a multigene family comprising four members in vertebrates that share a highly conserved DNA-binding domain capable of binding to M-CAT elements, which are found in the promoters of many genes expressed in cardiac and skeletal muscles (Jin *et al*., 2011). Many functions have been described for RTEF-1, including enhancement of the promoter activity of VEGF (Shie *et al*., 2004) and hypoxia-inducible factor-1α (HIF-1α) (Jin, 2011) under hypoxia, increased angiogenesis in the hindlimb ischaemia model (Shie *et al*., 2004; Jin, 2011), facilitation of communication between the endothelium and myocardium (Xu *et al*., 2011), enhancement of endothelial-dependent microvascular relaxation (Messmer-Blust *et al*., 2012a), regulation of cell-to-cell connections and aggregation via connexin 43, connexin 40, and connexin 37 (An *et al*., 2012) and attenuation of blood glucose levels through insulin-like growth factor binding protein-1 (IGFBP1) regulation in ECs (Messmer-Blust *et al*., 2012b). We hypothesized that RTEF-1 may be impaired during aging, as it serves as a regulatory factor during angiogenesis progression.

In this study, we explored variations in miRNA and RTEF-1 expression in cultured ECs collected from young and old thoracic aortas of C57BL/6 male mice. We found that miR-125a-5p was upregulated in parallel with the downregulation of RTEF-1 protein in old endothelial cells (OECs). In addition, we found that induction or repression of mir-125a-5p resulted in a decrease or increase in angiogenesis ability *in vitro*, and we established that these effects on angiogenesis involved the regulation of its target gene, RTEF-1.

## Materials and methods

### Animals

Male C57BL/6 mice aging 6 to 10 weeks (young) and 16 to 18 months (old) were purchased from the Sun Yat-sen University Laboratory Animal Centre. All mice were housed in sterile microisolator cages with a standard light–dark cycle, and they received a standard ultraviolet-treated diet along with autoclaved drinking water. Each mouse was anaesthetized by intraperitoneal injection of 0.3–0.4 mL pentobarbital sodium (10 mg/mL) (Kobayashi *et al*., 2005), and the thoracic aorta was collected. All animal procedures were approved by the Research Ethics Committee of the First Affiliated Hospital, Sun Yat-sen University.

### Cell culture

Arterial endothelial cells (ECs) were isolated from the thoracic aortas of C57BL6 male mice (A new batch of cells was isolated from a separate group of three mice for each experiment) and cultured in the EC-specific medium EGM-2 (Lonza, Switzerland) (Yagi *et al*., 2007) supplemented with 5% foetal serum at 37 °C in a humidified environment with 5% CO_2_. The cells were grown for 3–6 generations before use in the experiments. Immunohistochemical identification of ECs by factor VIII staining (Belloni & Tressler, 1990) showed that the samples were more than 90% pure.

### Microarray analysis

Arterial ECs were isolated from the thoracic aortas of young or aging C57BL6 male mice. Five groups were established with three mice per group, for a total of 15 young mice and 15 aging mice. Total RNA was isolated from the ECs by phenol–chloroform isolation (TRIzol, Invitrogen, CA, USA). RNA was pooled in equal amounts, and microarray analyses for all identified murine miRNAs (miRBase 9.0) were performed using the All-in-One™ miRNA qPCR Array (Exiqon, Denmark) (Sadoun & Reed, 2003).

### Small RNA transfection

ECs were cultured in EGM-2 medium and seeded into 6-well plates at a density of 2.5 × 10 (Ambros, 2004) cells/well. After overnight incubation, young endothelial cells (YECs) were transfected with 50 nm pre-miR-125a-5p, and OECs were transfected with 100 nm anti-miR-125a-5p or their respective negative controls (RiboBio, Guangzhou, China) using Lipofectamine™ RNAMiX (Invitrogen) according to the manufacturer’s instructions. After 12 h, the transfection medium was replaced with serum-free DMEM. Total RNA or protein samples were extracted using the TRIzol Reagent after 24 h (Invitrogen) or using RIPA buffer after 48 h, respectively, from both transfected and untransfected ECs (Zhu *et al*., 2013).

### Quantitative real-time PCR for mRNA expression determination

A total of 1.0 μg of RNA from transfected cells and negative controls was reverse-transcribed using the ReverTra Ace qPCR RT kit (TOYOBO, Osaka, Japan) or an miRNA reverse transcription kit (Takara, Dalian, China) according to the manufacturer’s instructions. Quantitative real-time PCR (qRT–PCR) was performed with specific primers (Table S1) using THUNDERBIRD™ SYBR qPCR mix (TOYOBO) or a SYBR PrimeScript™ miRNA RT–PCR kit (Takara, Dalian, China) according to the manufacturer’s recommendations. Real-time quantitative PCR was performed on a CFX96 System (Bio-Rad, Hercules, CA, USA). For each cDNA sample, amplification of specific mRNA or miRNA sequences was performed in at least three independent experiments. Relative expression levels were determined by the ∆∆Ct method using the housekeeping genes GAPDH and U6 for normalization (Zhu *et al*., 2013).

### Matrigel tube formation assay

The *in vitro* angiogenic activity of ECs was determined using the Matrigel tube formation assay as described previously (Heinke *et al*., 2008). Briefly, cells were plated at a density of 2 × 10 (Grundmann *et al*., 2011) cells/well in 96-well plates precoated with growth factor-reduced Matrigel (BD Biosciences, Bedford, MA, USA) according to the manufacturer’s instructions. After 8 h of incubation, tube formation, defined as the appearance of a circle-like structure, was observed under a light microscope (Olympus, Tokyo, Japan). Images of tube morphology were obtained in ten random microscopic fields per sample at 100× magnification, and the cumulative mean tube lengths per field of view (*n* = 5 images/condition) were quantified by imagej software.

### Spheroid sprouting assay

EC spheroids were generated as described previously (Korff & Augustin, 1998; Heinke *et al*., 2008). In brief, ECs were suspended in culture medium containing 0.2% carboxymethylcellulose (Sigma, St. Louis, MI, USA) 12 h after transfection and seeded in nonadhesive 96-well plates. The suspended cells formed a single spheroid in each well with a defined size and cell number (400 cells/spheroid). Spheroids were generated overnight; thereafter, they were embedded into collagen gels. Spheroid-containing gels were rapidly transferred into prewarmed 24-well plates and allowed to polymerize (30 min). Then, 100 μL of endothelial basal medium was added, and images were acquired using an OLYMPUS IX51 microscope after 24 h of incubation. *In vitro* capillary sprouting was quantified by measuring the cumulative length using XV image processing software (Olympus). The mean cumulative sprout length per spheroid was calculated based on the examination of 10–15 spheroids.

### 3′-UTR luciferase assay

The 3′UTR luciferase assay was performed as previously described (Boon *et al*., 2011). Fragments of the 3′UTR of RTEF-1 containing putative or mutated miR-125a-5p binding sites were amplified by RT–PCR using specific primers (Table S2). According to the manufacturer’s instructions, the products were cloned into pmir-RB-REPORT™ vectors (Ribio Biotech, Guangzhou, China) downstream from the hR-luc luciferase coding sequence. The constructs were cotransfected with pre-mir-125a-5p or a negative control (pre-mir-125a-5p-NC) into 3T3 cells using Lipofectamine 2000 (Invitrogen). After 48 h of transfection, luciferase activity was measured using the Dual-Luciferase Reporter Assay System Kit (Promega Biotech, Madison, WI, USA). hR-luc activities were first normalized to the internal control (h-luc) to evaluate the transfection efficiency. All experiments were performed three times. mir-125a-5p-mediated repression of hR-luc/h-luc activity was calculated as the ratio of hR-luc/h-luc in miR-125a-5p-transfected cells to hR-luc/h-luc in control oligotransfected cells.

### siRNA experiments

Small interfering RNA (siRNA) sequences targeting mouse RTEF-1 (Figure 6A) were synthesized by RiboBio Co., Ltd. (Guangzhou, China). RTEF-1 siRNA or a negative control (siRNA-NC) was used at a final concentration of 50 nm according to the manufacturer’s instructions, and the cells were transfected for 24 or 48 h. Subsequently, the knock-down efficiency in OECs was determined by RT–PCR and Western blot assays. In addition, 100 nm anti-miR-125a-5p (RiboBio, Guangzhou, China) was introduced alone or in combination with 50 nm RTEF-1 SiRNA using Lipofectamine™ RNAiMAX (Invitrogen) in OECs. The spheroid angiogenesis assay was then performed as described previously (Messmer-Blust *et al*., 2012b).

### Western blot

For Western blot analyses of RTEF-1, eNOS and VEGF expression, cultured ECs were lysed in RIPA buffer on ice, and protein concentrations were determined using the Pierce BCA Protein Assay Kit (Thermo Scientific, Waltham, MA, USA). Equal amounts of protein (20 μg) were separated with 10% or 15% SDS-PAGE and transferred to polyvinylidene difluoride (PVDF) membranes (Millipore, Darmstadt, Germany). After blocking with TBST supplemented with 5% nonfat milk, the membranes were incubated overnight at 4 °C with primary antibodies targeted against mouse RTEF-1 (1:2000, Abcam, Cambridge, UK), eNOS (1:1000, BD Bioscience), VEGF (1:500; Santa Cruz Biotechnology, Santa Cruz, CA, USA) and GAPDH (1:1000; Cell Signaling Technologies, Beverly, MA, USA). Secondary antibodies conjugated to horseradish peroxidase were purchased from Cell Signaling Technologies. Detection was performed using Novex ECL (Invitrogen), and imagej software was used for signal quantification (Heinke *et al*., 2008).

### Statistics

All data are expressed as the mean ± SEM. Two treatment groups were compared using the independent samples t test, and three or more groups were compared by one-way analysis of variance (ANOVA) followed by post hoc analysis adjusted with a least significant difference correction for multiple comparisons (SPSS, Chicago, IL, USA). Differences with a *P*-value<0.05 were considered significant.

## Results

### Microarray results and mir-125a-5p expression

To assess the changes in the miRNA transcriptome between YECs and OECs, miRNA microarray analyses were performed with total RNA isolated from ECs. We established two screening indexes for microarray analyses: first, we filtered out miRNAs with low expression in ECs and a low fold change between YECs and OECs, and second, we applied a statistical threshold of *P* < 0.05 (ANOVA).

Confirming the validity of our screening technique, the top 10 upregulated and downregulated miRNAs included several miRNAs previously described to be involved in vascularization, proliferation, migration and apoptosis (Tables 1 and 2). In addition, upregulation of mir-26a, mir-29a and mir-23a (Boon *et al*., 2011; Dellago *et al*., 2013) has been reported in aging mice and humans. One of the miRNAs showing the highest increase was mir-34b-3p. Interestingly, the mir-34 family has been shown to induce growth arrest and apoptosis in cancer cells (Hermeking, 2010). However, hsa-mir-34b-3p was not found in searches of TargetScan (http://www.targetscan.org/) or Miranda (http://www.microrna.org/microrna/home.do).

**Table 1:** Top 10 upregulated miRNAs selected from highly expressed miRNAs in ECs

Mature_ID	Ct value (OEC)	Ct value (YEC)	FC (OEC/YEC)	Function
mmu-miR-34b-3p	23.26	28.19	7.69	Senescence
mmu-miR-199a-3p	19.43	19.57	2.17	Angiogenesis
mmu-miR-145	22.08	22.26	1.36	Differentiation
mmu-miR-199b	19.55	19.01	1.36	Angiogenesis
mmu-miR-199a-5p	21.25	21.42	1.35	Angiogenesis
mmu-miR-125a-5p	21.80	21.87	1.26	Inflammation
mmu-miR-26a	20.94	21.00	1.25	Aneurysms
mmu-miR-29a	19.39	18.72	1.24	Aneurysms
mmu-miR-1944	21.28	22.07	1.18	Unknown
mmu-miR-23a	22.03	21.24	1.15	Aging

FC, fold change; Ct, cycle threshold.

**Table 2:** Top 10 downregulated miRNAs selected from highly expressed miRNAs in ECs

Mature_ID	Ct value (OEC)	Ct value (YEC)	FC (OEC/YEC)	Function
mmu-miR-320	21.26	19.12	−3.66	Diabetes
mmu-miR-1965	22.14	21.24	−2.72	Unknown
mmu-miR-1274a	21.24	20.48	−2.48	Unknown
mmu-miR-1959	22.61	22.04	−2.16	Unknown
mmu-miR-689	22.02	20.50	−2.16	Unknown
mmu-miR-2143	20.05	19.54	−2.08	Unknown
mmu-miR-2138	22.76	22.31	−2.00	Unknown
mmu-miR-720	20.44	19.04	−1.98	Unknown
mmu-miR-2134	20.77	20.36	−1.94	Unknown
mmu-miR-2137	20.24	19.89	−1.86	Unknown

FC, fold change; Ct, cycle threshold.

In addition to the upregulated miRNAs, the top 10 downregulated miRNAs were not associated with any blood vessel-related function. However, mir-125a-5p, which was the most consistently upregulated miRNA and is highly conserved between mice and humans, had not been previously studied in the context of blood vessel growth. Therefore, we selected mir-125a-5p for further investigation; we performed qRT–PCR using poly-A primers for mir-125a-5p in YECs and OECs to confirm our microarray data.

**Figure 1:**
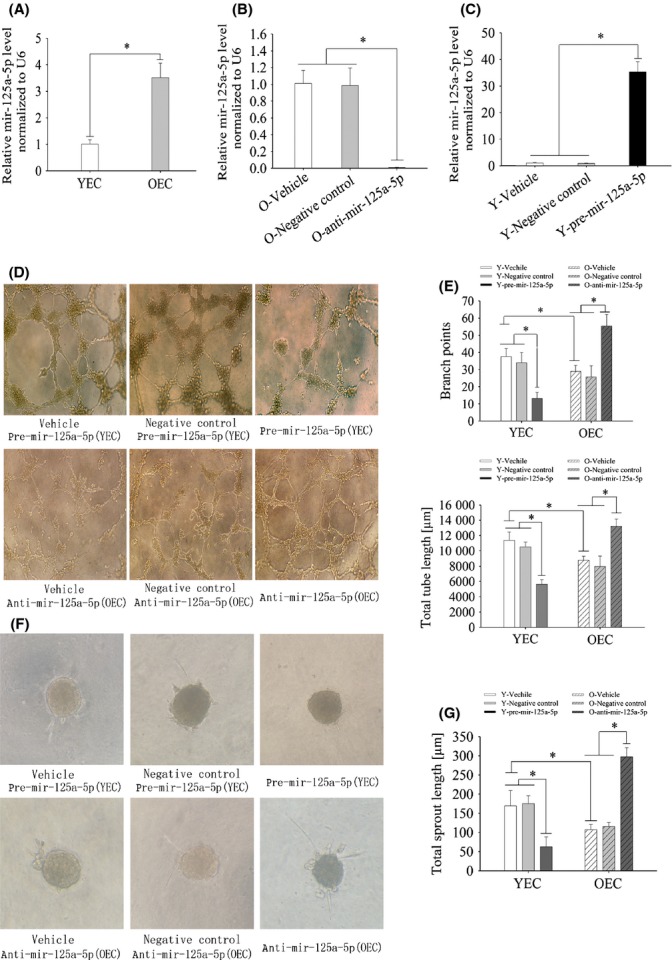
Mir-125a-5p expression in ECs and transfection with pre-mir-125a-5p or anti-mir-125a-5p regulates angiogenesis *in vitro*. A, qRT–PCR showed approximately 2.9-fold higher mir-125a-5p expression in OECs compared with young cells. B, qRT–PCR analysis of mir-125a-5p expression in OECs transfected with anti-mir-125a-5p; expression was normalized to U6 and is presented relative to vehicle control. C, qRT–PCR analysis of mir-125a-5p expression in YECs transfected with pre-mir-125a-5p; expression was normalized to U6 and is presented relative to vehicle control (A,B,C, *P < 0.05 versus YEC group, *n* = 3). D,E, transfected cells were cultured on Matrigel, and the cumulative sprout length of circle-like structures was measured after 8 h. MiR-125a-5p overexpression attenuated angiogenic tube formation in the Matrigel assay, whereas miR-125a-5p inhibition showed a stimulatory effect. F,G, transfected EC spheroids were embedded in collagen, and the cumulative sprout length was measured after 24 h. Pre-mir-125a-5p transfection inhibited endothelial sprouting, and anti-mir-125a-5p transfection had stimulatory effects. (Adobe ImageReady CS2 9.0 was used to adjust the contrast of the images.) The data represent the mean ± SEM.* *P* < 0.05 vs vehicle group, *n* = 5.

We found that mir-125a-5p expression showed an approximately threefold increase in OECs compared with YECs (Fig. 1A). Therefore, the initial screening results indicated that mir-125a-5p was abundant in ECs and significantly upregulated in OECs versus young cells.

### miR-125a-5p negatively regulates angiogenesis in vitro

To investigate whether mir-125a-5p functionally affects angiogenesis *in vitro*, we overexpressed or inhibited mir-125a-5p in ECs and assessed the subsequent changes using tube formation and three-dimensional spheroid sprouting assays. After 12 h, ECs were efficiently transfected with anti-miR-125a-5p or pre-miR-125a-5p, resulting in significant mir-125a-5p repression (Fig. 1B) or upregulation (Fig. 1C), respectively.

We modulated mir-125a-5p levels in ECs and subsequently performed a Matrigel assay. In this angiogenesis assay, mir-125a-5p overexpression in YECs inhibited tubule formation as shown by significant reductions in tube length (approximately 40%) and branch points (45%), whereas mir-125a-5p inhibition in OECs led to 30% and 45% increases in tube length and branch points, respectively (Fig. 1D, E). In addition, we found that mir-125a-5p overexpression in YECs blocked sprout formation, with a 50% decrease in total sprout length, whereas mir-125a-5p inhibition resulted in a 30% increase in tubular sprout outgrowth in OECs (Fig. 1F, G).

### RTEF-1 protein expression was reduced in OECs

**Figure 2:**
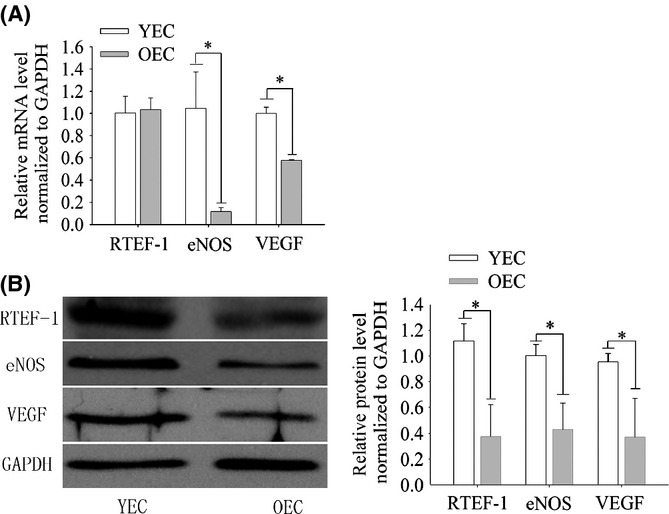
Protein and mRNA expression of RTEF-1, eNOS and VEGF in ECs. A, qRT–PCR analysis of RTEF-1, eNOS and VEGF expression. The results display a decrease in the mRNA levels of eNOS and VEGF but no change in RTEF-1 in OECs; expression was normalized to GAPDH and is presented relative to YECs. B, Western blot analysis of RTEF-1, eNOS and VEGF expression. The results display a decrease in the protein levels of RTEF-1, eNOS and VEGF; expression was normalized to GAPDH and is presented relative to YECs. The data represent the mean ± SEM. *P < 0.05 vs YECs, *n* = 3.

To further understand the mechanisms by which mir-125a-5p regulates angiogenesis, we searched for mir-125a-5p targets in databases using TargetScan and Miranda, and RTEF-1, a transcription factor involved in angiogenesis, was identified. RTEF-1 targets genes such as VEGF and HIF-1α in endothelial cells under hypoxia and increases vascular relaxation by modulating eNOS (Dellago *et al*., 2013; Zhu *et al*., 2013). In addition, previous studies have shown that VEGF promoter activity is reduced in aged animals (Rivard *et al*., 1999). We assessed and compared RTEF-1 gene and protein expression by qRT–PCR and Western blot assays, respectively, in both YECs and OECs. Our data showed no significant differences in RTEF-1 mRNA expression between the two cell groups (Fig. 2A). However, RTEF-1 protein expression was lower in OECs compared with YECs (Fig. 2B). Interestingly, the expression of VEGF and eNOS, which are downstream targets of RTEF-1, was reduced in OECs compared with YECs at both the mRNA (Fig. 2A) and protein (Fig. 2B) level.

### miR-125a-5p targets RTEF-1

**Figure 3:**
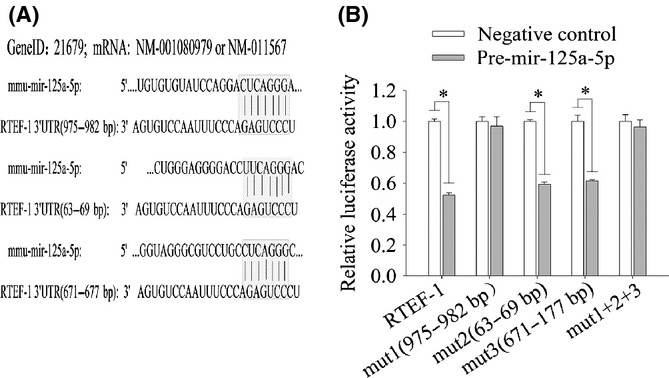
RTEF-1 is a target of mir-125a-5p. A, three predicted and conserved mir-125a-5p target sites in the 3′UTR of mouse RTEF-1. B, luciferase reporter gene analysis revealed the effect of cotransfecting a negative control (mir-125a-5p) and pre-mir-125a-5p with wild-type RTEF-1-pmir-reporter or RTEF-1-pmir-reporter with mutated target sites (site 1 [nucleotide positions 975-982], site 2 [positions 63-69], site 3 [position 671-177], or all three sites). Significance was measured compared with the negative control. The data represent the mean ± SEM. *P < 0.05 vs negative control, *n* = 3.

Based on the data described above, we hypothesized that RTEF-1 might be a direct target of mir-125a-5p. Indeed, we observed that the RTEF-1 3′UTR contained three conserved target sites (Fig. 3A), and RTEF-1 expression was significantly repressed after cotransfection with mir-125a-5p. Similar results were obtained when the second or third target site was mutated. Importantly, mutating all three target sequences or the first target site, which is complementary to the mir-125a-5p seed region, attenuated the repressive effect of mir-125a-5p on luciferase activity (Fig. 3B). Thus, mir-125a-5p-induced repression of RTEF-1 expression is dependent on the first target sequence.

Because miRNA targeting of mRNA results in translational blockage and mRNA degradation, we assessed RTEF-1 mRNA expression. However, we found no obvious change (Fig. 2A), demonstrating that regulation by miR-125a-5p occurs by translational repression rather than by mRNA synthesis/degradation.

### Upregulation of mir-125a-5p reduces angiogenic growth factor expression in YECs

As shown above, mir-125a-5p inhibits angiogenesis *in vitro*, and mir-125a-5p levels negatively correlate with RTEF-1 levels in ECs. In addition, using luciferase reporter assays, we demonstrated that RTEF-1 is a direct target of mir-125a-5p.

**Figure 4:**
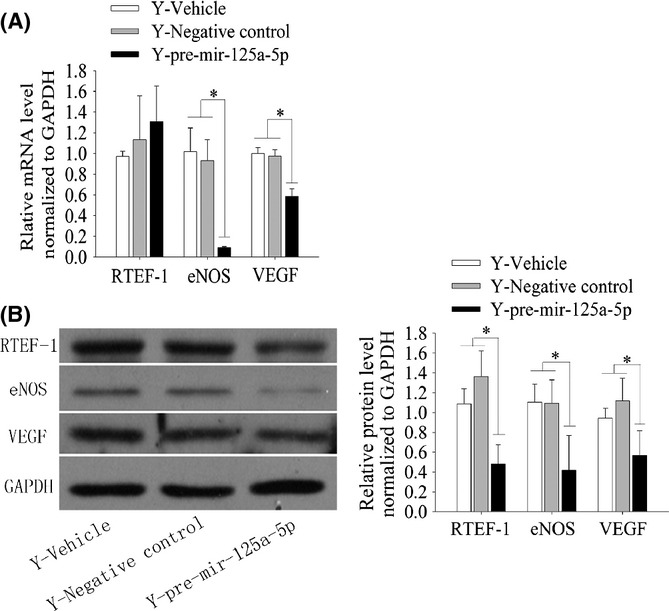
Mir-125a-5p negatively regulates the angiogenic signalling pathway by reducing angiogenic growth factor expression. A, qRT–PCR analysis of RTEF-1, eNOS and VEGF expression after overexpression of mir-125a-5p in YECs. The results reveal decreased expression of eNOS and VEGF but no change in RTEF-1; expression was normalized to GAPDH and is presented relative to the vehicle control. B, Western blot analysis of RTEF-1, eNOS, and VEGF expression after transfection of YECs with pre-mir-125a-5p. The results display a decrease in the protein levels of RTEF-1, eNOS and VEGF; expression was normalized to GAPDH and is presented relative to vehicle control. imagej software was used for quantification. The data represent the mean ± SEM. *P < 0.05 vs vehicle, *n* = 3.

To further explore the functional effect of mir-125a-5p and clarify the relationship between mir-125a-5p and RTEF-1, we overexpressed mir-125a-5p by transfecting pre-mir-125a-5p into YECs (Fig. 1B) and evaluated the expression of RTEF-1, eNOS and VEGF by qRT–PCR and Western blot assays. We found reduced levels of VEGF and eNOS mRNA in transfected YECs (Fig. 4A), and similar results were obtained for VEGF and eNOS protein levels (Fig. 4B). Although RTEF-1 protein expression also decreased (Fig. 4B), there were no significant differences in RTEF-1 mRNA levels between the cell groups (Fig. 4A).

These results confirm that mir-125a-5p downregulates the expression of RTEF-1 by post-transcriptional mechanisms rather than by altering mRNA synthesis/degradation in YECs.

### Downregulation of mir-125a-5p in OECs increases angiogenic growth factor expression

**Figure 5:**
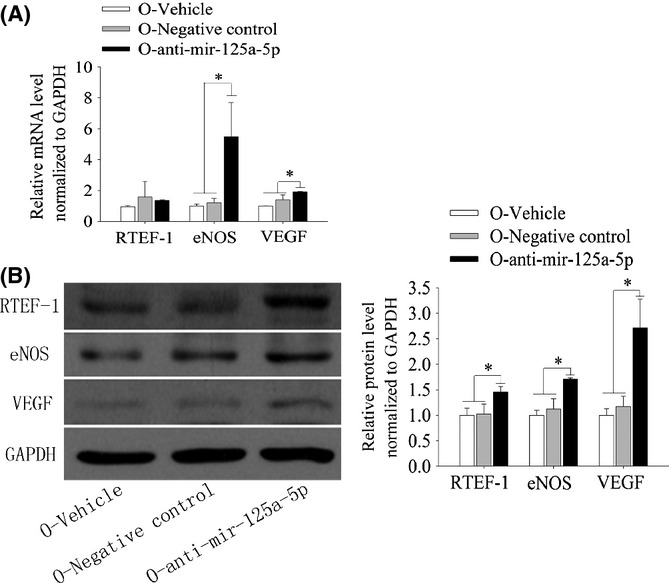
Anti-mir-125a-5p regulates the angiogenic signalling pathway by increasing the expression of angiogenic growth factors. A, qRT–PCR analysis of RTEF-1, eNOS and VEGF expression after repression of mir-125a-5p in OECs. The results display increased expression of eNOS and VEGF but no change in RTEF-1; expression was normalized to GAPDH and is presented relative to the vehicle control. B, Western blot analysis of RTEF-1, eNOS and VEGF expression after transfection of OECs with anti-mir-125a-5p; the results display an increase in the protein levels of RTEF-1, eNOS and VEGF; expression was normalized to GAPDH and is presented relative to the vehicle control. imagej software was used for quantification. The data represent the mean ± SEM. *P < 0.05 vs vehicle, *n* = 3.

Based on our previous results, we investigated whether mir-125a-5p downregulation increases RTEF-1 expression in OECs. Similar to previous experiments, we first evaluated the transfection efficiency (Fig. 1C) and determined the expression of RTEF-1, eNOS and VEGF. We found that mir-125a-5p downregulation resulted in increased VEGF and eNOS mRNA levels (Fig. 5A) in OECs; similar results were obtained for VEGF and eNOS protein levels (Fig. 5B). Although RTEF-1 protein levels also increased (Fig. 5B), there was no significant difference in RTEF-1 gene expression between the cell groups (Fig. 5A).

Taken together, these results show that changes in mir-125a-5p expression modulate eNOS and VEGF gene and protein levels as well as RTEF-1 protein levels in ECs. Because RTEF-1 promotes the expression of eNOS and VEGF by binding their promoters in ECs, we hypothesized that mir-125a-5p might affect angiogenesis through the regulation of VEGF and eNOS expression via RTEF-1.

### RTEF-1 knock-down inhibits angiogenesis

**Figure 6:**
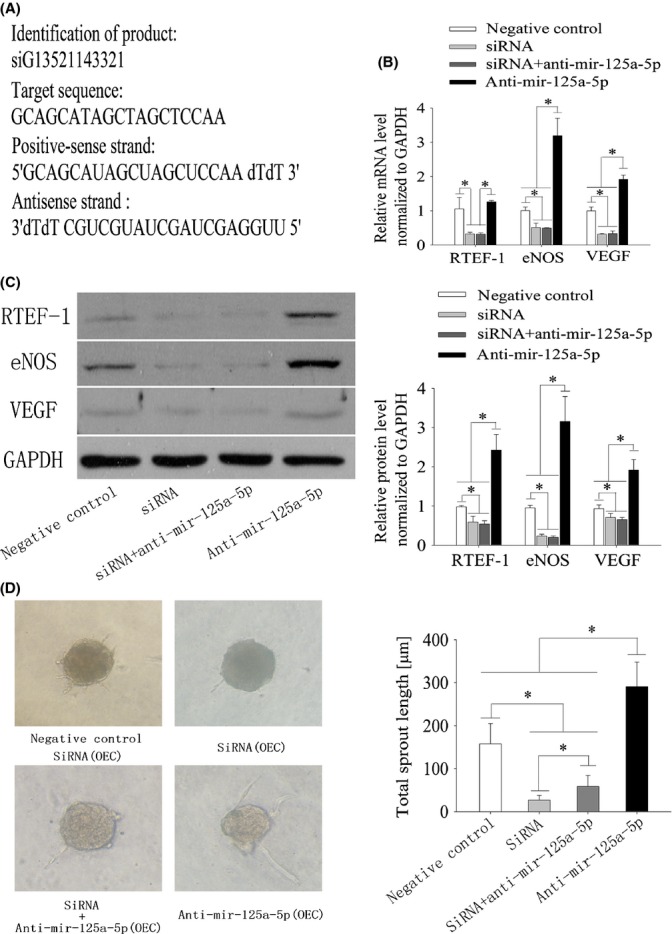
RTEF-1 knock-down inhibits OEC sprouting and reduces RTEF-1, VEGF and eNOS expression at the mRNA and protein levels. A, the product information of siRNA against RTEF-1. B, qRT–PCR analysis of RTEF- 1, VEGF and eNOS expression after transfection of OECs with siRNA-NC, RTEF-1 siRNA, siRNA+anti-mir-125a-5p and anti-mir-125a-5p. The results display decreased expression of RTEF-1, eNOS and VEGF in the siRNA and siRNA+anti-mir-125a-5p groups and increased expression of eNOS and VEGF but not RTEF-1 in the anti-mir-125a-5p group. Expression was normalized to GAPDH and is presented relative to siRNA-NC. C, Western blot analysis of RTEF-1, VEGF and eNOS expression in OECs transfected with negative control siRNA, RTEF-1 siRNA, siRNA+anti-mir-125a-5p and anti-mir-125a-5p. The results display decreased expression of RTEF-1, eNOS and VEGF in the siRNA and siRNA+anti-mir-125a-5p groups and increased expression of RTEF-1, eNOS and VEGF in the anti-mir-125a-5p group. Expression was normalized to GAPDH and is presented relative to siRNA-NC. D, spheroid angiogenesis assay of OECs transfected with siRNA-NC, RTEF-1 siRNA or anti-mir-125a-5p and cotransfected with RTEF-1 siRNA+anti-mir-125a-5p. The results reveal that RTEF-1 siRNA repressed EC sprouting, anti-mir-125a-5p promoted EC sprouting, and cotransfection of OECs with both RTEF-1 siRNA+anti-mir-125a-5p could not completely rescue the negative effect of RTEF-1 downregulation on EC sprouting relative to siRNA-NC. The data represent mean ± SEM. *P < 0.05 vs the vehicle group, *n* = 5.

To investigate whether mir-125a-5p exerts its antiangiogenic effects via RTEF-1, we knocked down RTEF-1 in OECs using siRNA (Fig. 6A–C) and subsequently performed spheroid angiogenesis assays. RTEF-1 silencing had a similar effect on angiogenesis to that observed with mir-125a-5p overexpression. An approximately fourfold decrease in sprout length was observed in siRNA-transfected OECs and a 35% increase in outgrowth of tubular sprouts in anti-mir-125a-5p-transfected OECs compared with siRNA-NC-transfected OECs. Cotransfection of OECs with both RTEF-1 siRNA and anti-mir-125a-5p did not rescue the repressive effect of RTEF-1 downregulation on angiogenesis, as indicated by a significant decrease in tubular sprouting (Fig. 6D). It has previously been shown that RTEF-1 deficiency results in decreased VEGF and eNOS expression. VEGF and eNOS are regulators of angiogenesis, and we have demonstrated the effect of RTEF-1 knock-down on angiogenesis (Fig. 6C). Therefore, our data confirm that mir-125a-5p directly regulates angiogenesis by modulating the expression of RTEF-1, which in turn regulates its downstream targets VEGF and eNOS.

## Discussion

Angiogenesis, which is the development of new vessels from pre-existing vasculature, is a complex process that includes activation, migration and proliferation of endothelial cells (D’Amore & Thompson, 1987). Recent studies have demonstrated that angiogenesis is progressively retarded during the aging process. Endothelial cell dysfunction; reduced expression of VEGF, eNOS, and FGF; increased expression of TSP-1 play important roles in this phenomenon (Rivard *et al*., 1999; Sadoun & Reed, 2003). miRNAs negatively regulate genes via degradation or translational inhibition of their target mRNAs (Ambros, 2004), and increasing evidence supports the role of miRNAs in ECs as a common molecular mechanism underlying impaired angiogenesis in aged animals (Menghini *et al*., 2009; Zhao *et al*., 2010; van Mil *et al*., 2012). In addition, miRNAs are expressed in a time-dependent manner; therefore, we assumed that miRNA expression levels differ between young and old ECs, which may contribute to impaired angiogenesis during aging.

Recent studies have suggested important regulatory roles for miRNAs such as miR-21, miR-216, miR-217, miR-181b, miR-31b and miR-34a, which were confirmed to be upregulated in senescing HUVECs (Menghini *et al*., 2009), and miR-146, miR-142-3p, miR-223 and miR-29 family members, which were significantly increased in whole aortas of aged mice (Zhao *et al*., 2010). However, these aging-related miRNAs are also found in aged HUVECs and circulating endothelial progenitor cells, and they can be induced by ischaemia. Therefore, the expression profiles and functions of miRNAs in ECs from healthy elderly animals are not fully understood.

We isolated ECs in young and aging mice under normal physiological conditions. Using gene array analysis, we found that mir-125a-5p expression was 1.26 times higher, while mir-34b-3p expression was 7.69 times higher in OECs compared with YECs. However, qRT–PCR analysis indicated that mir-125a-5p expression was 2.9 times higher in OECs than in YECs, and the sequence of mir-125a-5p is highly conserved between mice and humans. We could not detect has-mir-34b-3p using TargetScan software, and we were unable to identify potential targets of mmu-mir-34b-3p related to angiogenesis. Therefore, we explored the function of mir-125a-5p in ECs.

mir-125a-5p is downregulated in many cancers (de Oliveira *et al*., 2012; Sand *et al*., 2012), which strongly suggests that mir-125a-5p functions as a suppressor of proliferation and migration in cancer cells. In noncancer cells, mir-125a-5p mediates lipid uptake, decreases the secretion of inflammatory cytokines (IL-2, IL-6 and TNF-α) and the expression of oxysterol binding protein-like-9 (ORP9) in human monocytic cells (Chen *et al*., 2009), and suppresses ET-1 expression in human umbilical vascular endothelial cells (HUVECs) (Li *et al*., 2010). Interestingly, mir-125a-5p was shown to reduce the expression of VEGF and MMP11 in hepatocellular carcinoma cells (Bi *et al*., 2012). However, IL-2, IL-6, TNF-α, ORP9 and ET-1 were not found to be targets of mir-125a-5p based on a search of the TargetScanMouse database. Because EC migration and proliferation are important for angiogenesis and mir-125a-5p regulates these functions in human cancer cells, we hypothesized that mir-125a-5p plays a role in angiogenesis via similar regulatory mechanisms, causing reduced angiogenesis ability in aging mice.

We demonstrated that mir-125a-5p has an antiangiogenic effect based on spheroid sprouting and tube formation assays in ECs. Next, we aimed to elucidate the mechanisms underlying the effect of mir-125a-5p on angiogenesis via analysis of its predicted targets in the database. Previous studies have reported the downregulation of certain OEC proteins that regulate angiogenesis including VEGF, eNOS, FGF-2 and PDGF (Phillips & Stone, 1994; Rivard *et al*., 1999; Swift *et al*., 1999; Sadoun & Reed, 2003). We found decreased expression levels of VEGF and eNOS in OECs compared with YECs at both the gene and protein levels. However, their mRNAs were not found to be mir-125a-5p targets in the database. It is plausible that mir-125a-5p indirectly regulates VEGF and eNOS expression, similar to the mir-221/222 mechanism that regulates eNOS in ECs (Rippe *et al*., 2012).

Interestingly, RTEF-1, a transcription factor that regulates VEGF and eNOS expression in ECs, was identified in the database as a potential mir-125a-5p target. Our data showed that RTEF-1 protein levels were lower in OECs than in YECs, indicating that RTEF-1 is a potential target of mir-125a-5p and most likely plays a key role in mir-125a-5p regulation of angiogenesis. To further explore the relationship between mir-125a-5p and RTEF-1, mir-125a-5p expression was modulated by transfecting YECs with pre-mir-125a-5p or OECs with anti-mir-125a-5p. After overexpression or repression of mir-125a-5p in ECs, we found that the protein expression of VEGF and eNOS correlated with RTEF-1 expression. Although VEGF and eNOS mRNA levels were decreased or increased in transfected YECs or OECs, respectively, there were no obvious changes in RTEF-1 mRNA levels in the different cell groups. This result indicated that RTEF-1 is regulated by mir-125a-5p at the post-transcriptional level, although VEGF and eNOS are regulated by mir-125a-5p at the transcriptional level. Previous studies have suggested that a decrease in VEGF promoter activity is the cause of the VEGF reduction observed in old vascular smooth muscle cells (VSMCs) compared with young cells (Rivard *et al*., 1999). Taken together, we propose that mir-125a-5p directly regulates the expression of RTEF-1, which in turn regulates VEGF and eNOS expression, contributing to the decreased angiogenic ability of ECs.

Using a dual-luciferase reporter assay, we confirmed that nucleotide positions 975-982 in the 3′UTR of RTEF-1 are a direct target of mir-125a-5p. To test whether mir-125a-5p exerts its antiangiogenic effect by targeting RTEF-1, we knocked down RTEF-1 using siRNA in OECs and evaluated angiogenesis using spheroid sprouting assays. In agreement with the data described above, we demonstrated that angiogenesis defects were associated with RTEF-1 deficiency, as reflected by vascular sprouting defects after mir-125a-5p overexpression or RTEF-1 knock-down. Interestingly, RTEF-1 siRNA repressed EC sprouting, while anti-mir-125a-5p promoted EC sprouting. However, cotransfection of OECs with both RTEF-1 siRNA and anti-mir-125a-5p could not completely rescue the negative impact of RTEF-1 downregulation on EC sprouting. These results suggest that the effects on EC angiogenesis result mostly from mir-125a-5p inhibition of RTEF-1. Therefore, mir-125a-5p may have other modes of action by which it regulates angiogenesis aside from its effect on RTEF-1. However, at present, we lack evidence from *in vivo* experiments; thus, our next step is to investigate the function of mir-125a-5p *in vivo*.

In conclusion, an important role for mir-125a-5p in angiogenesis regulation via RTEF-1 was elucidated for the first time *in vitro*. Our findings suggest that RTEF-1 has a protective role against the aging process and subsequent vasculopathy. Although many technical aspects and safety issues remain to be resolved, mir-125a-5p and RTEF-1 targeting could constitute a new therapeutic tool for use in cardiovascular diseases.

## Supporting Information

**Table S1.** Primer sequences for qRT-PCR. **Table S2.** Primer sequences for 3’UTR Luciferase assay.

## References

[b1] Ambros V (2004). The functions of animal microRNAs. Nature.

[b2] An X, Jin Y, Philbrick MJ, Wu J, Messmer-Blust A, Song X, Cully BL, He P, Xu M, Duffy HS, Li J (2012). Endothelial cells require related transcription enhancer factor-1 for cell-cell connections through the induction of gap junction proteins. Arterioscler. Thromb. Vasc. Biol.

[b3] Belloni PN, Tressler RJ (1990). Microvascular endothelial cell heterogeneity: interactions with leukocytes and tumor cells. Cancer Metastasis Rev.

[b4] Bi Q, Tang S, Xia L, Du R, Fan R, Gao L, Jin J, Liang S, Chen Z, Xu G, Nie Y, Wu K, Liu J, Shi Y, Ding J, Fan D (2012). Ectopic expression of MiR-125a inhibits the proliferation and metastasis of hepatocellular carcinoma by targeting MMP11 and VEGF. PLoS ONE.

[b5] Boon RA, Seeger T, Heydt S, Fischer A, Hergenreider E, Horrevoets AJ, Vinciguerra M, Rosenthal N, Sciacca S, Pilato M, van Heijningen P, Essers J, Brandes RP, Zeiher AM, Dimmeler S (2011). MicroRNA-29 in aortic dilation: implications for aneurysm formation. Circ. Res.

[b6] Carmeliet P (2000). Mechanisms of angiogenesis and arteriogenesis. Nat. Med.

[b7] Chen T, Huang Z, Wang L, Wang Y, Wu F, Meng S, Wang C (2009). MicroRNA-125a-5p partly regulates the inflammatory response, lipid uptake, and ORP9 expression in oxLDL-stimulated monocyte/macrophages. Cardiovasc. Res.

[b8] D’Amore PA, Thompson RW (1987). Mechanisms of angiogenesis. Annu. Rev. Physiol.

[b9] Dellago H, Preschitz-Kammerhofer B, Terlecki-Zaniewicz L, Schreiner C, Fortschegger K, Chang MW, Hackl M, Monteforte R, Kuhnel H, Schosserer M, Gruber F, Tschachler E, Scheideler M, Grillari-Voglauer R, Grillari J, Wieser M (2013). High levels of oncomiR-21 contribute to the senescence-induced growth arrest in normal human cells and its knock-down increases the replicative lifespan. Aging Cell.

[b10] Grundmann S, Hans FP, Kinniry S, Heinke J, Helbing T, Bluhm F, Sluijter JP, Hoefer I, Pasterkamp G, Bode C, Moser M (2011). MicroRNA-100 regulates neovascularization by suppression of mammalian target of rapamycin in endothelial and vascular smooth muscle cells. Circulation.

[b11] Heinke J, Wehofsits L, Zhou Q, Zoeller C, Baar KM, Helbing T, Laib A, Augustin H, Bode C, Patterson C, Moser M (2008). BMPER is an endothelial cell regulator and controls bone morphogenetic protein-4-dependent angiogenesis. Circ. Res.

[b12] Hermeking H (2010). The miR-34 family in cancer and apoptosis. Cell Death Differ.

[b13] Jin YWJSX (2011). RTEF-1, an Upstream Gene of Hypoxia-inducible Factor-1a, Accelerates Recovery from Ischemia. J. Biol. Chem.

[b14] Jin Y, Messmer-Blust AF, Li J (2011). The role of transcription enhancer factors in cardiovascular biology. Trends Cardiovasc. Med.

[b15] Kobayashi M, Inoue K, Warabi E, Minami T, Kodama T (2005). A simple method of isolating mouse aortic endothelial cells. J. Atheroscler. Thromb.

[b16] Korff T, Augustin HG (1998). Integration of endothelial cells in multicellular spheroids prevents apoptosis and induces differentiation. J. Cell Biol.

[b17] Li D, Yang P, Xiong Q, Song X, Yang X, Liu L, Yuan W, Rui YC (2010). MicroRNA-125a/b-5p inhibits endothelin-1 expression in vascular endothelial cells. J. Hypertens.

[b18] Menghini R, Casagrande V, Cardellini M, Martelli E, Terrinoni A, Amati F, Vasa-Nicotera M, Ippoliti A, Novelli G, Melino G, Lauro R, Federici M (2009). MicroRNA 217 modulates endothelial cell senescence via silent information regulator 1. Circulation.

[b19] Messmer-Blust AF, Zhang C, Shie JL, Song Q, He P, Lubenec I, Liu Y, Sellke F, Li J (2012a). Related transcriptional enhancer factor 1 increases endothelial-dependent microvascular relaxation and proliferation. J. Vasc. Res.

[b20] Messmer-Blust AF, Philbrick MJ, Guo S, Wu J, He P, Guo S, Li J (2012b). RTEF-1 attenuates blood glucose levels by regulating insulin-like growth factor binding protein-1 in the endothelium. Circ. Res.

[b21] van Mil A, Grundmann S, Goumans MJ, Lei Z, Oerlemans MI, Jaksani S, Doevendans PA, Sluijter JP (2012). MicroRNA-214 inhibits angiogenesis by targeting Quaking and reducing angiogenic growth factor release. Cardiovasc. Res.

[b22] de Oliveira JC, Scrideli CA, Brassesco MS, Morales AG, Pezuk JA, Queiroz RP, Yunes JA, Brandalise SR, Tone LG (2012). Differential miRNA expression in childhood acute lymphoblastic leukemia and association with clinical and biological features. Leuk. Res.

[b23] Phillips GD, Stone AM (1994). PDGF-BB induced chemotaxis is impaired in aged capillary endothelial cells. Mech. Aging Dev.

[b24] Rippe C, Blimline M, Magerko KA, Lawson BR, LaRocca TJ, Donato AJ, Seals DR (2012). MicroRNA changes in human arterial endothelial cells with senescence: relation to apoptosis, eNOS and inflammation. Exp. Gerontol.

[b25] Rivard A, Fabre JE, Silver M, Chen D, Murohara T, Kearney M, Magner M, Asahara T, Isner JM (1999). Age-dependent impairment of angiogenesis. Circulation.

[b26] Sadoun E, Reed MJ (2003). Impaired angiogenesis in aging is associated with alterations in vessel density, matrix composition, inflammatory response, and growth factor expression. J. Histochem. Cytochem.

[b27] Sand M, Skrygan M, Sand D, Georgas D, Hahn SA, Gambichler T, Altmeyer P, Bechara FG (2012). Expression of microRNAs in basal cell carcinoma. Br. J. Dermatol.

[b28] Shie JL, Wu G, Wu J, Liu FF, Laham RJ, Oettgen P, Li J (2004). RTEF-1, a novel transcriptional stimulator of vascular endothelial growth factor in hypoxic endothelial cells. J. Biol. Chem.

[b29] Swift ME, Kleinman HK, DiPietro LA (1999). Impaired wound repair and delayed angiogenesis in aged mice. Lab. Invest.

[b30] Xu M, Jin Y, Song Q, Wu J, Philbrick MJ, Cully BL, An X, Guo L, Gao F, Li J (2011). The endothelium-dependent effect of RTEF-1 in pressure overload cardiac hypertrophy: role of VEGF-B. Cardiovasc. Res.

[b31] Yagi R, Kohn MJ, Karavanova I, Kaneko KJ, Vullhorst D, DePamphilis ML, Buonanno A (2007). Transcription factor TEAD4 specifies the trophectoderm lineage at the beginning of mammalian development. Development.

[b32] Zhao T, Li J, Chen AF (2010). MicroRNA-34a induces endothelial progenitor cell senescence and impedes its angiogenesis via suppressing silent information regulator 1. Am. J. Physiol. Endocrinol. Metab.

[b33] Zhu S, Deng S, Ma Q, Zhang T, Jia C, Zhuo D, Yang F, Wei J, Wang L, Dykxhoorn DM, Hare JM, Goldschmidt-Clermont PJ, Dong C (2013). MicroRNA-10A* and MicroRNA-21 modulate endothelial progenitor cell senescence via suppressing high-mobility group A2. Circ. Res.

