# Supplementary material for: miR-125a-5p impairs endothelial cell angiogenesis in aging mice via RTEF-1 downregulation
Source: Aging Cell. 2014 Jul 24;13(5):926–34. doi: 10.1111/acel.12252 (PMC4331751; doi:10.1111/acel.12252)
Supplement: Supplementary file 1 — Table S1. Primer sequences for qRT-PCR. Table S2. Primer sequences for 3’UTR Luciferase assay. [file acel0013-0926-sd1.docx]

| RTEF-1 | F:5’-ACAATGATGCAGAGGGTGTATG-3’ | R:5’-TCCTCCGTCAGGATAATTTTGC-3’ |
| --- | --- | --- |
| eNOS | F:5’-ATAGCCCGCATAGCGTATCAG-3’ | R:5’-TCAGCCATCACAGTGTTCCC-3’ |
| VEGF | F:5’-GCACATAGAGAGAATGAGCTTCC-3’ | R:5’-CTCCGCTCTGAACAAGGCT-3’ |
| GAPDH | F:5’-AGGTCGGTGTGAACGGATTTG-3’ | R:5’-TGTAGACCATGTAGTTGAGGTCA-3’ |

Table 1

| m-RTEF-1 -3UTR-F | 5'-CCGCTCGAGGAGACTTGGAGAGCAATGGTAGG-3' |
| --- | --- |
| m-RTEF-1-3UTR-R | 5'-GAATGCGGCCGCAGTACCAATGCATATTTATTGAACATGTG-3' |
| m-RTEF-1 -3UTR-mu1-F968 | 5'-TCCAGGAGTGACCCATGCATCTGTGAGGATG-3' |
| m-RTEF-1-3UTR-mut1-R990 | 5'-CAGATGCATGGGTCACTCCTGGATACACACAA-3' |
| m-RTEF-1-3UTR-mut2-F56 | 5'-GGGACCTTGACCCACAGTCCCCTGGAAGTG-3' |
| m-RTEF-1-3UTR-mut2-R76 | 5'-GGGACTGTGGGTCAAGGTCCCCTCCCAGG-3' |
| m-RTEF1-3UTR-mut3-F664 | 5'-GTCCTGCGTGACCCCCAGCATTTCCAGCTG-3' |
| m-RTEF-1-3UTR-mut3-R685 | 5'-AATGCTGGGGGTCACGCAGGACGCCCTACC-3' |

Table 2
